# An Economical Bioprocess for Lipopeptide Production From *Bacillus subtilis*

**DOI:** 10.1002/mbo3.70130

**Published:** 2026-05-08

**Authors:** Derya Maral‐Gül, Zülal Günay, Baran Mis, Rengin Eltem

**Affiliations:** ^1^ Department of Bioengineering, Graduate School of Natural and Applied Sciences Ege University Bornova Izmir Turkey; ^2^ Department of Bioengineering, Faculty of Engineering Ege University Bornova Izmir Turkey

**Keywords:** antifungal effect, *Bacillus subtilis*, lipopeptid, production optimization, RSM

## Abstract

Phytopathogenic fungi such as *Fusarium solani* (*F. solani*), *Rhizoctonia solani* (*R. solani*), and *Botrytis cinerea* (*B. cinerea*) cause plant diseases, leading to significant yield losses. Lipopeptides (LPs), secondary metabolites produced by *Bacillus* strains, exhibit antifungal properties against these pathogens. In this study, the LP production potential of *Bacillus* isolates was investigated, and the best‐performing *Bacillus* sp. was selected for the optimization of the production medium and parameters. Hemolytic activities of *Bacillus* isolates were screened using blood agar, revealing that all 69 isolates exhibited hemolytic activity. The isolates were cultivated in Lysogeny Broth (LB) medium, and LP extractions were performed. The antifungal activity of LP extracts against *F. solani*, *R. solani*, and *B. cinerea* was assessed using the agar well diffusion method. The iturin and surfactin content of LP extracts was analyzed by quadrupole time‐of‐flight mass spectrometry. Five *Bacillus* sp. with high LP production potential were selected, and their cell growth was examined. Among them, *Bacillus subtilis* 4‐Ka‐22 demonstrated the highest cell growth. The cost‐effective optimized production medium was determined as 0.1% molasses, 1% soybean meal, 0.5% CaCl_2_, and 1% glycerol, with production parameters set at 30°C, 195 rpm, pH 5, and a 4.3% inoculum ratio. Compared to LB medium, cell growth increased by 13.4‐fold, confirming successful optimization.

## Graphical abstract

The lipopeptide (LP) production potential of *Bacillus* isolates was evaluated against phytopathogenic fungi, with *B. subtilis* 4‐Ka‐22 identified as the top‐performing strain. Optimization of the production medium—comprising molasses, soybean meal, CaCl_2_, and glycerol—resulted in a 13.4‐fold increase in cell growth and over 99% cost reduction compared to LB medium, demonstrating strong potential for large‐scale, cost‐effective LP production.

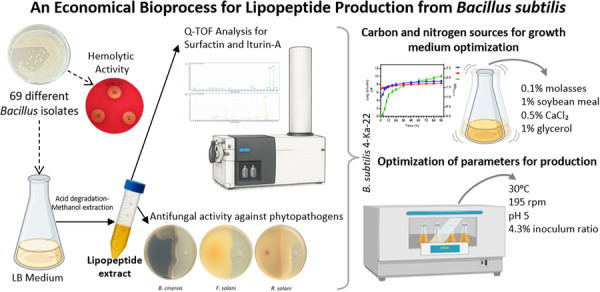

## 1 Introduction

*Bacillus* strains, known as plant growth promoting rhizobacteria (PGPR), are bacteria used in different fields thanks to their ability to produce various metabolites and form endospores (Stamenkovic‐Stojanovic et al. 2019). *Bacillus* sp. are considered one of the main sources of biological control agents (BCAs) due to their many valuable properties, especially endospore formation (Cawoy et al. 2011; Stamenkovic‐Stojanovic et al. 2019). Besides their endospore‐forming properties, *Bacillus* spp. not only tolerate harmful environmental conditions, but also manage to survive aggressive processing steps during large‐scale productions.

Fungi that cause disease and harvest loss in plants are called phytopathogenic fungi. *Botrytis cinerea* (*B. cinerea*), *Fusarium solani* (*F. solani*), and *Rhizoctonia solani* (*R. solani*) are the most common phytopathogenic fungi. *B. cinerea* is a filamentous fungal pathogen (van Kan 2005). It causes economic losses due to gray mold disease in the production of grapevine, tomato, strawberry, cucumber, cut flowers, bulbous flowers, and potted plants. *Fusarium* is a pathogen of many plants, a mycotoxin producer, and an opportunistic human pathogen. As a plant pathogen, *Fusarium* spp. cause fruit, root, and stem rot (Rampersad 2020). *Fusarium* diseases include wilts, diseases, rots, and cancers of many garden, field, ornamental, and forest crops in both agricultural and natural ecosystems (Ma et al. 2013). *R. solani* is a soil‐borne phytopathogen capable of affecting members of various plant families, including Poaceae (maize, rice, wheat, barley, oat), Fabaceae (soybean, peanut, common bean, alfalfa, chickpea, lentil, pea), Solanaceae (tobacco, potato), Amaranthaceae (sugar beet), Brassicaceae (canola), Rubiaceae (coffee), Malvaceae (cotton), Asteraceae (lettuce), Araceae (pothos), Moraceae (ficus), and Linaceae (flax) (Ajayi‐Oyetunde and Bradley 2018).

Lipopeptides (LPs), which are among the secondary metabolites produced by *Bacillus* sp., are known to show antifungal activity against these phytopathogenic fungi (Inès and Dhouha 2015; Toral et al. 2018; Ghazala et al. 2022). LPs contain amino acids and fatty acid chains in their structure. They are divided into three types as cyclic, partially cyclic, and linear LPs according to the state of the amino acid chains in their structures (Fewer et al. 2021). Many *Bacillus* spp., especially *Bacillus subtilis* (*B. subtilis*), have the ability to synthesize different LPs such as surfactin and iturin, which have cyclic LP structures (H. Zhao et al. 2017; Geissler et al. 2019). Thanks to the peptide and fatty acid chains in their structures, LPs with amphiphilic properties can exhibit high surface active – biosurfactant and antimicrobial properties (Patel et al. 2015; Geissler et al. 2019) and thus can be used in many different fields.

Surfactin is a cyclic heptapeptide composed of a C13–C15 fatty acid chain linked to a ring of seven amino acids. Due to its antibacterial, antiviral, and antifungal properties, it has broad applications (Carolin et al. 2021). Although surfactins exhibit relatively weak antifungal activity, they are of particular interest due to their ability to induce systemic resistance in host plants (Dame et al. 2021). They are effective against various plant diseases, including leaf blight, root and stem rot, leaf spot, and soft rot (Saxena et al. 2020). Iturins, another group of cyclic heptapeptides, consist of several variants such as iturin A and C, bacillomycins D, F, L, and mycosubtilin. These compounds vary in amino acid composition and fatty acid chain length (C14–C17) (Carolin et al. 2021). Iturins display strong antifungal activity against pathogens like *Penicillium*, *Aspergillus*, *Fusarium*, and *Botrytis*, though their antibacterial efficacy is limited.

Laboratory‐ and pilot‐scale production of LPs is achievable using various microorganisms. Lysogeny broth (LB) medium is commonly used to investigate LP production by *Bacillus* spp. (Meena et al. 2018; Sarwar et al. 2018; Soussi et al. 2019). Production processes may vary depending on the type of LP to be produced, its properties, the raw material to be used as medium, and the production volume (Geissler et al. 2019). Stress in production processes can increase LP production (Coutte et al. 2017). Various substrates such as cashew apple juice (de Oliveira et al. 2013), orange peels (Kumar et al. 2016), rapeseed meal (Jin et al. 2014), two‐phase olive mill waste (Maass et al. 2016), and cassava flour (Nitschke and Pastore 2004) can be used in LP production for economic benefit (Geissler et al. 2019).

In our previous study, the antifungal activity of 1574 *Bacillus* isolates against *B. cinerea*, *F. solani*, and *R. solani* was assessed using the dual culture assay. Following this screening, 69 *Bacillus* isolates that exhibited activity against these phytopathogens were selected. The selected isolates were further analyzed for their lytic enzyme activities (protease, chitinase, and chitosanase) and antifungal metabolite production (siderophores and hydrocyanic acid—HCN) (Maral‐Gül and Eltem 2024). In the present study, the LP production potential of these 69 *Bacillus* isolates was investigated. Additionally, using the selected *Bacillus* sp., an economically viable production medium formulation was determined, and the production parameters were optimized.

## 2 Experimental Procedures

### 2.1 *Bacillus* Isolates and Phytopathogenic Fungi

A total of 69 *Bacillus* isolates, 22 of which were molecularly identified, were obtained from the *Bacillus* culture collection of Ege University, Department of Bioengineering (Maral‐Gül and Eltem 2024). Nutrient Broth (NB) and Nutrient Agar (NA) were used for the production and storage of *Bacillus* spp. Phytopathogenic fungi *B. cinerea*, *F. solani*, and *R. solani* were obtained from the Manisa Viticulture Research Institute Directorate. Phytopathogenic fungi were activated on Potato Dextrose Agar (PDA) at 28°C for 5 days.

### 2.2 Hemolytic Activity Screening

*Bacillus* isolates, which were activated in NA for 24 h, were inoculated in three spots on petri dishes containing commercially available blood agar medium and incubated at 30°C for 72 h (Sarwar et al. 2018). The diameters of the formed transparent zones were measured. Human pathogen *Staphylococcus aureus* (*S. aureus*) BAA‐40, known for forming a zone of inhibition, was used as a positive control.

### 2.3 Lipopeptide Production in LB Medium and Extraction

LB (Oxoid, CM0996) was used for LP production. Pure colonies of freshly activated *Bacillus* isolates were transferred and incubated in NB for 24 h at 30°C and then they were transferred to sterile LB with 2% inoculation rate. Production was carried out in a shaker incubator at 30°C and 150 rpm for 96 h (Dimkić et al. 2017).

Acid degradation‐methanol extraction method was used to extract LPs. At the end of the incubation period, liquid culture was centrifuged at 9000 rpm for 15 min. The pH of the supernatant obtained after centrifugation was adjusted to 2 using 6 N HCl and the samples were stored at +4°C overnight. After the waiting period, the samples were centrifuged at 9000 rpm for 15 min to obtain pellets (Dimkić et al. 2017; Sarwar et al. 2018). The pellets were stored at +4°C overnight by adding 2 mL of methanol. The samples were centrifuged at 9000 rpm for 10 min to collect the methanol phase (Smyth et al. 2010; Lin et al. 2020). The extract obtained was sterilized by passing through a 0.45 μm pore diameter nylon syringe filter and stored at −20°C.

### 2.4 Determination of Antifungal Properties of LP Extracts by Agar Well Diffusion

Agar well diffusion was used to determine the effect of LP extracts against *F. solani*, *R. solani*, and *B. cinerea*. Fungal disks (6 mm) from activated phytopathogen colonies were placed on PDA plates. Wells (6 mm) were created 5 mm from the disks using sterile glass rods, and each well received 100 μL of LP extract. Petri dishes without LP extract were used as a control. Petri dishes containing *B. cinerea* and *F. solani* were incubated at 30°C for 10 days and those containing *R. solani* for 5 days. The antifungal effect of the LP extracts was determined in terms of percent growth inhibition (PGI) value, calculated according to Equation 1 (Orberá Ratón et al. 2012; Meena et al. 2018).

(1) Growth Inhibition(%)=[(R−r)/R]∗100

where *R* = diameter of the phytopathogen fungus in the control petri dish and *r* = diameter of phytopathogenic fungus growth against LP extract.

The PGI values calculated according to Equation (1) are divided into four categories and graded into growth inhibition category (GIC). 0: no growth inhibition, 1: 1%–25% growth inhibition, 2: 26%–50% growth inhibition, 3: 51%–75% growth inhibition, and 4: 76%–100% growth inhibition (Maral‐Gül and Eltem 2024).

### 2.5 Identification of Lipopeptide Extracts by Q‐TOF LC/MS

Quadrupole time‐of‐flight mass chromatography (Q‐TOF) analysis was performed using Agilent 6550 Q‐TOF LC/MS to identify the surfactin and iturin contents of the LP extracts obtained after extraction. Standards of surfactin ≥ 98% (CAS No. 24730‐31‐2) and iturin A ≥ 95% (CAS No. 52229‐90‐0) were purchased from Sigma‐Aldrich (Favaro et al. 2016; Toral et al. 2018; Wu et al. 2019).

### 2.6 Screening of *Bacillus* sp. and Growth Kinetics

The inoculum prepared in NB was transferred to LB medium with 2% inoculation rate. The inoculated LB media were incubated in a shaker incubator at 150 rpm and 30°C for 5 days. The number of cells was determined as colony‐forming units/mL (cfu/mL) by pour plate (Yezza et al. 2004).

In order to determine the growth kinetics of the *Bacillus* sp., which was found to show high antifungal activity and cell growth, 2% inoculation was made into NB using the inoculum prepared in LB medium. The medium was incubated at 180 rpm and 30°C for 4 days. During 96 h‐incubation period, samples were taken at 3‐h intervals under aseptic conditions. Cell growth was analyzed by plate count method, OD (optical density) was measured, and LP extractions were performed.

### 2.7 Carbon and Nitrogen Sources for Growth Medium Optimization

Glucose, fructose, sucrose, corn starch, potato extract, molasses (50% sugar content), whey, lactose, maltose, inulin, Tween 20, Tween 80, rice flour, glycerol, waste oil, and olive oil were added to the LB as a carbon source for the development of the selected *Bacillus* sp. and LP production. In order to determine the nitrogen source, peptone, soybean meal, yeast extract, meat extract, NH_4_Cl, urea, tryptone, (NH_4_)_2_SO_4_, distillers dried grain with solubles (DDGS), sodium glutamate, KNO_3_, NaNO_3_, and corn soaking liquid were added to the LB medium. MgSO_4_, MgCl_2_, MnSO_4_, MnCl_2_, FeSO_4_, FeCl_3_, CuSO_4_, CoCl_2_, CaCl_2_, ZnSO_4_, KI, NaCl, EDTA, H_3_BO_3_, H_3_PO_4_, Na_2_HPO_4_, K_2_HPO_4_, and KH_2_PO_4_ were added to the LB to determine other components to be used in the production medium. All carbon, nitrogen sources and other components used in the experiments were added to the LB at a 1% (w/v) ratio (equivalent to 0.5 g in 50 mL of medium).

After selecting the carbon and nitrogen sources to be used, Design Expert software 13, Box–Behnken analysis was performed to determine the amount of components. The minimum and maximum reference values to be used were determined as 0.1%–1% for carbon source, 1%–10% for nitrogen source, and 0.5%–1% for other medium components. A set of 17 experiments was obtained. The cell growth (cfu/mL) and antifungal effect (PGI value) were analyzed as predicted response. During the trials, the selected bacteria were grown on NB medium and inoculated into the production medium at 2%. Incubation was carried out in a shaker incubator at 30°C, 180 rpm for 72 h. As a result of the production, LP was extracted from the medium by the acid degradation method and antifungal activity against *F. solani* was determined by agar well diffusion method.

After determining the amounts of carbon, nitrogen, and other nutrients, 1%, 2%, 5%, and 10% glycerol was added to the medium to determine the amount of glycerol to be added to the production medium as a protective component and the productions were carried out under the same conditions. The amount of glycerol to be added to the production medium was determined by examining the cell growth and antifungal activity against *F. solani* by agar well diffusion method.

### 2.8 Optimization of Media Parameters for Production

After deciding on the content of the production medium, temperature, pH, rpm, and inoculation rate were examined to decide on the production parameters. To determine the temperature value, productions were carried out at 30°C, 33°C, 36°C, and 40°C for 3 days with 2% inoculation rate and 180 rpm shaker speed. The best temperature value for production was determined by measuring the cell growth and antifungal activity against *F. solani* in the samples taken during the incubation period.

Box–Behnken analysis for response surface methodology (RSM) was performed in Design Expert to optimize the pH, rpm, and inoculation rate parameters while keeping the temperature constant. During the optimization experiment, 150, 180, and 210 rpm values were used for shaker speed, 5.0, 7.0, and 9.0 values for pH, 1%, 3%, and 5% values for inoculation rate. A set of 17 experiments was obtained. After their production, cell growth and antifungal activity against *F. solani* were analyzed.

### 2.9 Statistical Replication

For the PGI assays against different phytopathogens, LP extracts obtained from three independent production batches were tested in triplicate, resulting in nine replicates per pathogen. Hemolytic activity, viable cell counts, and Q‐TOF analyses were conducted in triplicate.

## 3 Results and Discussion

### 3.1 Determination of Hemolytic Activity of *Bacillus* Isolates

Hemolytic activity assay can be used as a preliminary screening test for the isolation and identification of biosurfactant‐producing bacteria (Greber et al. 2018; Sarwar et al. 2018). In the hemolytic activity test, the formation of a transparent zone on blood agar medium indicates that the biosurfactant LPs produced by the bacteria can hemolyze blood (Sabino et al. 2024). Among the 69 *Bacillus* isolates examined in our study, 27 showed low hemolytic activity (9–13 mm), 26 showed medium hemolytic activity (13–17 mm), and 16 showed high hemolytic activity. To provide a positive control for hemolytic activity and facilitate reproducibility, the human pathogenic strain *S. aureus* BAA‐40, which is known for its strong hemolytic capacity, was included for comparison. *S. aureus* BAA‐40 formed an inhibition zone of 12 ± 1 mm in diameter on blood agar medium. Among the *Bacillus* isolates screened, 60 of them showed a higher hemolytic effect than *S. aureus* BAA‐40 by forming a diameter higher than 12 mm zone (Figure S1). However, this hemolytic effect was attributed to the LP production of the isolates since *Bacillus* isolates are not pathogenic. Besides their amphiphilic nature, LPs possess the ability to interact with lipid bilayers, which enables them to disrupt cell membranes and cause hemolysis of human erythrocytes (Ali et al. 2022).

### 3.2 Determination of Antifungal Effects of *Bacillus* Isolates Against Phytopathogens

In order to observe the growth of phytopathogenic *B. cinerea*, *F. solani*, and *R. solani* fungi, the fungi were cultivated in petri dishes containing PDA medium and the radius values were measured on the 5th and 10th days. Radii of 4.2 cm, 5.5 cm, and 6.6 cm were recorded for *B. cinerea*, *F. solani*, and *R. solani*, respectively.

**Figure 1:**
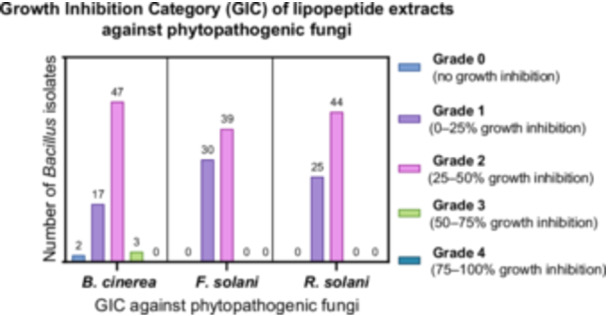
GIC values of LP extracts obtained from *Bacillus* isolates against *B. cinerea*, *F. solani*, and *R. solani*, distribution of the number of isolates.

The 69 *Bacillus* isolates used in the study were grown on LB medium, and LP extractions were carried out in the production medium. To examine the antifungal effects of the obtained LP extracts against *B. cinerea*, *F. solani*, and *R. solani*, antifungal activity analysis was performed by agar well diffusion method. After the analyses, the zone diameters were measured on Day 10 for *B. cinerea* and *F. solani* petri dishes and on Day 5 for *R. solani* petri dishes and PGI values were calculated according to Equation (1). GIC categories were determined using the calculated PGI values. The GIC values of 69 *Bacillus* spp. screened for their LPs against phytopathogenic fungi are shown in Figure 1.

**Figure 2:**
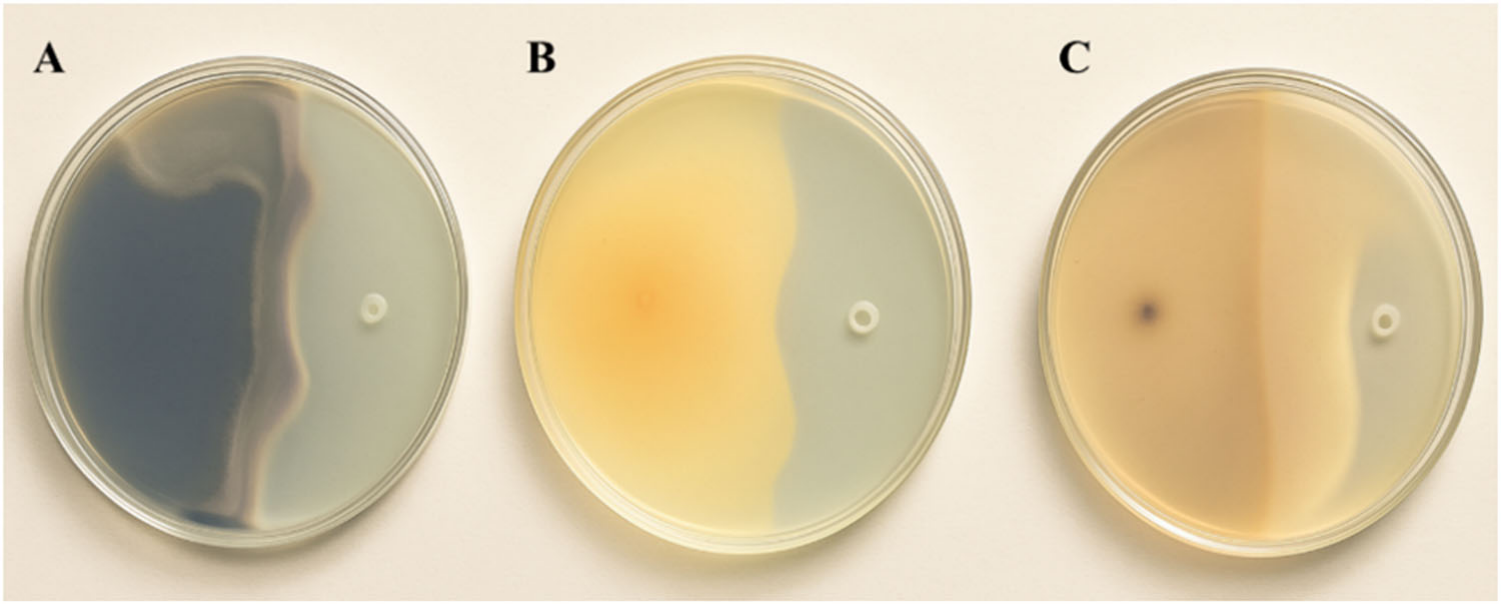
Effect of LP extract obtained from *B. subtilis* 2‐K‐29 against phytopathogenic fungi. (A) Effect against *B. cinerea* after 10 days incubation. (B) Effect against *F. solani* after 10 days incubation. (C) Effect against *R. solani* after 5 days incubation.

The highest inhibition zone against *B. cinerea* was 60.0% ± 6.2%, observed with the LP extract of *B. subtilis* 3‐K‐S‐64. Against *F. solani* (36.4% ± 0.3%) and *R. solani* (37.9% ± 0.7%), the strongest inhibition was exhibited by *Bacillus* isolate C‐3‐15‐a. Overall, the isolates demonstrated higher GIC values against *B. cinerea*. *Bacillus* isolate 3‐K‐S‐49, *B. subtilis* 3‐K‐S‐64, and 2‐K‐29 exhibited pronounced antifungal activity against phytopathogenic fungi. According to the GIC values of *Bacillus* isolates, the most effective isolate was *B. subtilis* 2‐K‐29 with 51% (GIC: 3) inhibition against *B. cinerea*, 35.2% (GIC: 2) against *F. solani,* and 27.8% (GIC) against *R. solani*. Agar well diffusion plates of *B. subtilis* 2‐K‐29 are shown in Figure 2. In addition, *Bacillus* isolates coded C‐3‐22‐a, C‐3‐22‐b, C‐3‐23, C‐3‐31, O‐1‐85, O‐3‐30, O‐4‐13‐a, O‐4‐57‐a, T‐3‐12‐b, T‐4‐20‐e‐a, C‐3‐20‐a, K‐7‐14‐1, 1‐K‐49‐a, and 3‐K‐S‐1‐a showed 2nd order GIC against all three phytopathogens. In general, the *Bacillus* isolates used showed higher inhibition effect against *B. cinerea*.

The antifungal effect of LP extracts obtained from *Bacillus* isolates grown on LB medium was compared to their performance in dual culture assays (Maral‐Gül and Eltem 2024). LP extracts exhibited lower PGI values than the dual culture method, likely due to additional antifungal metabolites produced during cocultivation. In a study evaluating 30 *Bacillus* isolates against *Rhizoctonia bataticola* (*R. bataticola*) and *Fusarium oxysporum (F. oxysporum) f. sp. ciceri*, isolate CaB5 showed the highest inhibition, with 53.3% and 58.5% inhibition in dual culture assays, respectively. However, crude LP extracts of the same isolate yielded lower inhibition rates (40% against *R. bataticola* and 41.6% against *F. oxysporum*) in agar well diffusion tests (Smitha et al. 2015).

### 3.3 Identification of Lipopeptide Extracts by Q‐TOF LC/MS

Surfactin (m/z 1036) and iturin A (m/z 1043) contents of the LP extracts obtained using the acid degradation‐methanol method were quantitatively analyzed by Q‐TOF LC/MS. Among the 69 *Bacillus* isolates screened, 68 were found to produce surfactin and/or iturin A at varying concentrations. Of these, 59 isolates produced iturin A and 65 produced surfactin (Table S1).

**Figure 3:**
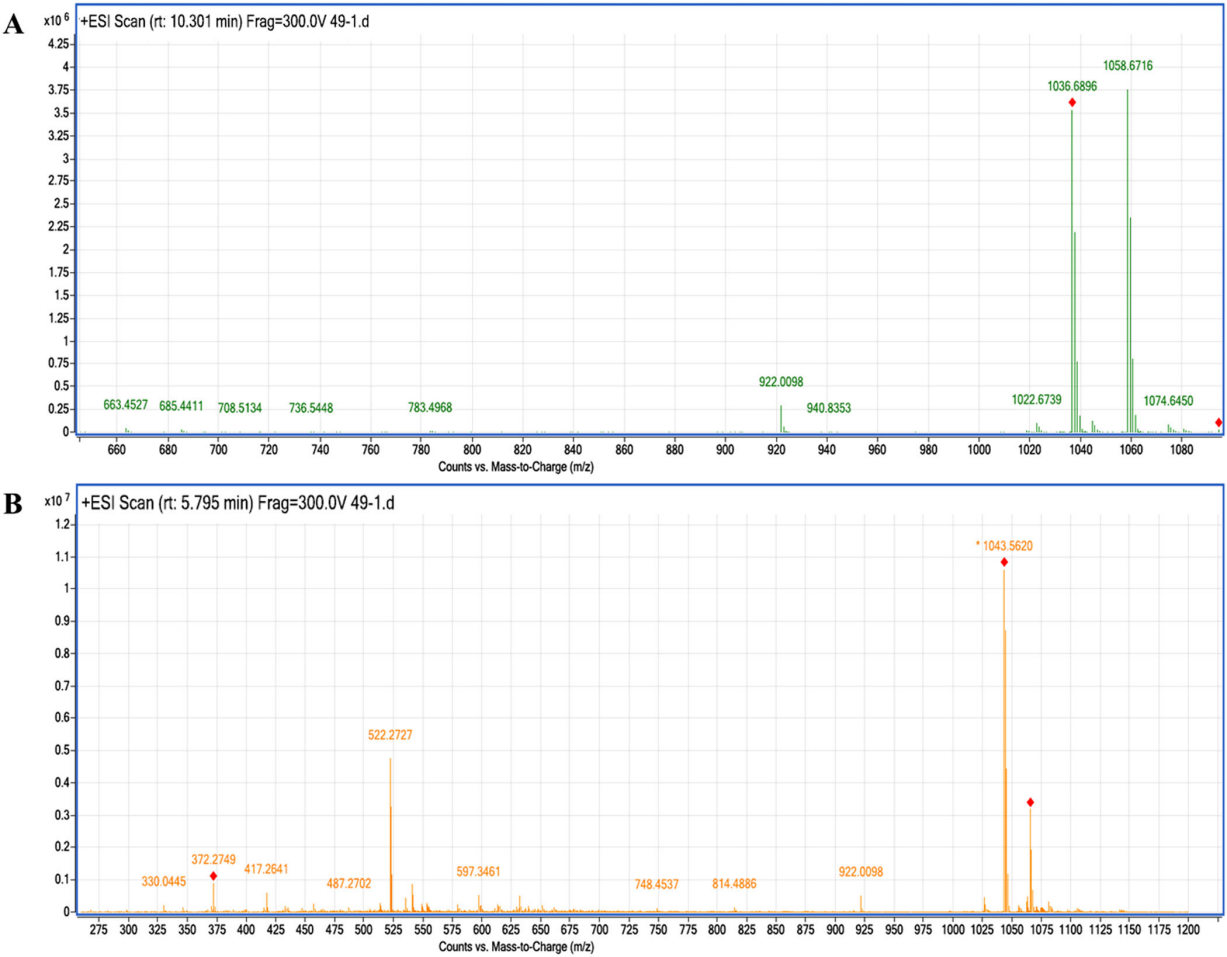
(A) Surfactin chromatogram for *B. subtilis* 4‐Ka‐22. (B) Iturin A chromatogram for *B. subtilis* T‐4‐14‐a.

Analysis of 69 screened *Bacillus* isolates revealed average production rates of 18.20 ± 11.83 μg/mL for iturin A and 15.56 ± 11.45 μg/mL for surfactin. The high standard deviations detected in the average LP amounts are evidence that different *Bacillus* isolates show different effects on LP production. The minimum and maximum amounts of surfactin determined in 68 *Bacillus* isolates determined to produce iturin A and surfactin ranged between 3.06 and 41.9 μg/mL, while the amounts of iturin A ranged between 0.1 and 45.39 μg/mL. The highest amount of surfactin was 41.9 ± 3.5 μg/mL in isolate 4‐Ka‐22 and the highest amount of iturin A was 45.39 ± 13.0 μg/mL in isolate T‐4‐14‐a. The surfactin chromatogram for the highest surfactin producing *B. subtilis* 4‐Ka‐22 and the iturin A chromatogram for the highest iturin A producing *B. subtilis* T‐4‐14‐a are shown in Figure 3.

*Bacillus* 3‐K‐S‐14 isolate, which was found not to synthesize surfactin and iturin A, showed activity against phytopathogenic fungi when examined by agar well diffusion method. It is thought that this effect may be due to the production of fengycin, another LP type. As a result of the Q‐TOF LC/MS analysis performed within the scope of the studies, *B. subtilis* sp. with high LP production potential were identified as K‐9‐37‐a, 3‐K‐S‐64, 4‐Ka‐22, 4‐Ka‐59‐a‐a, and C‐2‐30‐b. Although all isolates produced LPs, the differences observed in their antimicrobial spectrum and hemolytic activity likely stem from variations in both the qualitative make‐up and relative abundance of LP families and their various isoforms (Isaia et al. 2025). Additionally, physicochemical properties such as the length of the fatty acid chain, peptide sequence, and net charge substantially influence interactions with microbial membranes, thereby modulating biological activity (Guillén‐Navarro et al. 2023).

### 3.4 Selection of *B. subtilis* and Determination of Growth Curve

In the continuation of the study, to select the *B. subtilis* strain to be used for production medium optimization, biomass production was also examined in addition to the above‐mentioned analyses. As a result of Q‐TOF LC/MS analysis, five different *B. subtilis* with high LP production (K‐9‐37‐a, 3‐K‐S‐64, 4‐Ka‐22, 4‐Ka‐59‐a‐a, and C‐2‐30‐b) were selected. The selected strains were cultivated on LB medium and incubated for 5 days to enable detailed monitoring of *Bacillus* growth. Upon completion of the production phase, 45 mL samples were collected for subsequent analyses. Cell growth was determined in cfu/mL by the pour plate method. The data of five different *B. subtilis* selected for biomass production are given in Table 1. Using SPPS package program and Tukey's test, it was determined that the *Bacillus* sp. reached the same amount of live cell counts logarithmically and there was no statistically significant difference (*p* < 0.05) between them.

**Table 1:** Hemolytic activity (mm), PGI and GIC values against phytopathogens, amounts of surfactin and iturin produced (μg/mL), and cell growth (cfu/mL) of five different *B. subtilis* tested for biomass production.

*Bacillus* sp.	Hemolytic activity (mm)	PGI/(GIC)			Q‐TOF/LC‐MS		Cell growth (×10^8^ cfu/mL)
		*B. cinerea*	*F. solani*	*R. solani*	Surfactin (μg/mL)	Iturin A (μg/mL)	
*B. subtilis* K‐9‐37‐a ON722555	21	% 34.2 (2)	% 23.5 (1)	% 29.7 (2)	40.1 ± 2.6	32.7 ± 0.7	3.71 ± 1.45^a^
*B. subtilis* 3‐K‐S‐64 ON722562	13	% 60 (3)	% 24.6 (1)	% 33.1 (2)	9.43 ± 2.2	15.82 ± 0.5	3.37 ± 2.23^a^
*B. subtilis* 4‐Ka‐22 ON722556	18	% 33.1 (2)	% 24.1 (1)	% 25 (1)	41.9 ± 3.5	31.9 ± 0.4	2.44 ± 0.62^a^
*B. subtilis* 4‐Ka‐59‐a‐a ON722564	17	% 45.9 (2)	% 30.8 (2)	% 23.3 (1)	8.6 ± 0.4	38.15 ± 6.5	4.04 ± 0.88^a^
*B. subtilis* C−2‐30‐b ON722558	13	% 11.9 (1)	% 32.1 (2)	% 29.3 (2)	10.93 ± 2.9	28.99 ± 0.2	1.66 ± 0.56^a^

^a^ Means within a column with the same letter are not significantly different according to Tukey's test (*p* < 0.05).

LP production is recognized as one of the key mechanisms underlying the antifungal activity of PGPRs used as BCAs. Based on LP production levels, *B. subtilis* 4‐Ka‐22 was identified as the highest producer of surfactin and iturin A among the 69 screened strains and was selected for further optimization studies involving production media and parameters.

**Figure 4:**
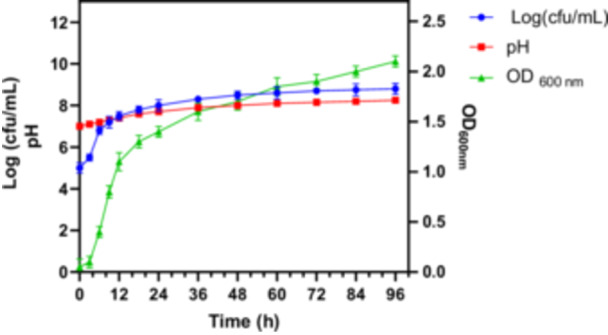
Growth curve of *B. subtilis* 4‐Ka‐22 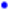 cell growth (Log(cfu/ml)) 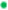 OD 600 nm absorbance and 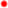 pH.

In order to examine the growth of *B. subtilis* 4‐Ka‐22, a growth curve was constructed using pour plate results in LB liquid medium, with LP extractions conducted at defined time intervals. The initial pH of the LB medium was 7.1, rising to 9.5 by the end of the cultivation period. Inoculation of LB medium with a preculture grown in NB medium led to a short lag phase, followed by exponential growth until the 9th hour, after which a second lag phase was observed. Cell growth and OD_600_ values increased again after the 18th hour, peaking around the 72nd hour before declining. This diauxic growth pattern may be attributed to the sequential utilization of tryptone and yeast extract in the LB medium, or to the depletion of a self‐produced metabolite. The growth curve (log CFU/mL), OD, and pH values of *B. subtilis* 4‐Ka‐22 are presented in Figure 4.

**Figure 5:**
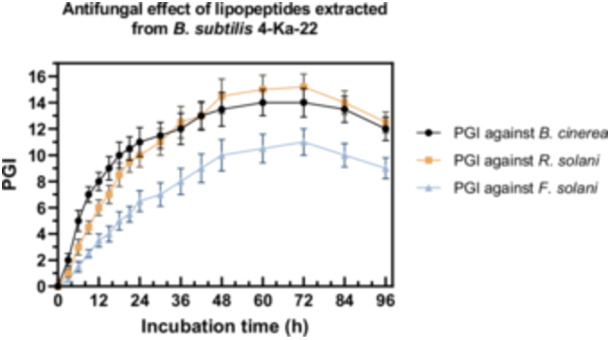
Antifungal effect of lipopeptides extracted from *B. subtilis* 4‐Ka‐22 against ● *B. cinerea*, 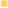 *R. solani* and 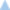 *F. solani*.

LPs produced by *Bacillus* sp. are produced depending on the growth state of the microorganism. Surfactin production starts in the logarithmic phase and continues until the stationary phase (Ayed et al. 2019). Iturin A production starts at the end of the logarithmic phase and the production of iturin derivatives continues throughout the incubation period (Coutte et al. 2017; Ayed et al. 2019). LP extracts collected at various time points from the production medium were tested for antifungal activity against *B. cinerea*, *F. solani*, and *R. solani*. For each analysis, 45 mL of the production medium was used, from which 4 mL of methanol extract was obtained after acid precipitation. The antifungal activity trend was consistent with the bacterial growth kinetics, reaching a maximum at 72 h, followed by a decline. Consequently, the optimal incubation period was determined to be 72 h. The antifungal activity profile of the extracts is presented in Figure 5.

X. Zhao et al. (2014) reported that when *Bacillus* sp. BH072 was cultivated in LB medium, the logarithmic phase occurred between 12 and 18 h, and maximum antifungal activity was observed at 60 h. Similarly, in a study with *B. subtilis* N7, cell growth was shown to precede LP production, with LP accumulation beginning during the stationary phase, particularly between 36 and 60 h (Luo 2013). The highest LP yield was recorded at 60 h, followed by a 30% decline due to cell lysis between 72 and 84 h. Additionally, the antifungal efficacy of LPs was found to decrease when the pH of the production medium exceeded 9.0 (Magdalena et al. 2020; Wang et al. 2020).

### 3.5 Optimization of Growth Medium and Parameters

The choice of carbon source can significantly influence both microbial growth and secondary metabolite production. To improve the medium performance, various carbon, nitrogen, and additional components were incorporated into LB medium. Media supplemented with specific components resulted in higher cell density and PGI values compared to unsupplemented LB. Olive oil, Tween 80, and molasses enhanced cell growth, while the highest antifungal activity of LP extracts was observed in media containing corn starch and lactose (Figure S2). Considering both PGI values and production cost, molasses was selected as the preferred carbon source for further studies.

Nitrogen source type and concentration also impacted metabolite production (Pournejati and Karbalaei‐Heidari 2020). Soybean meal, DDGS, tryptone, and yeast extract all supported cell growth (Figure S3). Although peptone and yeast extract yielded the highest antifungal activity, soybean meal was chosen for its comparable efficacy, superior growth support, and lower cost.

Screening of additional components revealed that CaCl_2_ promoted cell growth, while Na_2_HPO_4_, KH_2_PO_4_, KI, and CaCl_2_ positively influenced PGI values (Figure S4). As no significant PGI differences were observed among these, CaCl_2_ was selected. In contrast, FeCl_3_, ZnSO_4_, CoCl_2_, and EDTA inhibited *B. subtilis* 4‐Ka‐22 growth.

The potential of carbon sources, nitrogen sources, and other components were added to the LB medium and whose effects on cell growth and PGI were examined to form a production medium were added to the LB medium and examined alone. The variables of the experimental design and the results obtained after the growth of *B. subtilis* 4‐Ka‐22 are given in Table 2. After the addition of different concentrations of the components, normal LB medium (no:8) with 1% of each component was used as a control group. When live cell counts and PGI values were analyzed as a result of the experiments, it was determined that the number of live cells and PGI ratio were higher in the medium containing 1% molasses, soybean, and CaCl_2_ (no:1) compared to the medium (no:8) formed by adding these components to LB medium. Considering the data, it was determined that molasses, soybean meal, and CaCl_2_ compounds can be used to create a cost‐effective production medium without LB liquid medium.

**Table 2:** Examination of the components decided to be used in the production medium.

	Production medium (%)			Results	
No.	Molasses	Soybean meal	CaCl_2_	Cell growth ± SD^a^ (×10^7^ kob/mL)	PGI against *F. solani* ± SD (%)
1	1%	1%	1%	171 ± 32^a^	15.0 ± 1.11^b^
2	1%	10%	1%	2.06 ± 0.29^b^	9.44 ± 2.12^e^
3	5%	10%	1%	3.45 ± 0.35^b^	10.0 ± 1.28^d,e^
4	1%	10%	0.50%	2.62 ± 0.42^b^	13.33 ± 0^b,c^
5	5%	10%	0.50%	5.56 ± 0.54^b^	13.33 ± 1.81^c,d^
6	10%	10%	0.50%	8.02 ± 0.24^b^	12.22 ± 1.28^b,c^
7	10%	10%	1%	11.13 ± 0.65^b^	13.33 ± 1.81^b,c^
8	LB + 1%	LB + 1%	LB + 1%	163.33 ± 34.5^a^	7.78 ± 1.28^e^
Control	LB medium			42.3 ± 0.35^b^	23.33 ± 1.28^a^

*Note:* Means within columns with the same letter are not significantly different according to Tukey's test (*p* < 0.05).

^a^ SD, standard deviation.

Increasing the amount of molasses used as a carbon source did not show a logarithmically significant effect on the cell growth, while the highest biomass was obtained at low molasses concentrations. Therefore, it was decided to use minimum 0.1% and maximum 1% molasses in the RSM trial. When soybean meal was used in low amounts, an increase in the number of viable cells was observed, while the percentage growth inhibition value was higher when soybean meal was used in high amounts. Therefore, it was decided to use 1% and 10% soybean meal as minimum and maximum values in the RSM experiment.

### 3.6 RSM Optimization of Production Medium Components

For the optimization of the quantities of the components in the production medium, RSM analysis and Box–Behnken experimental design were decided to be used. As a result of the experimental design, a 17‐experiment design was obtained. The cell growth obtained as a result of the production of *B. subtilis* 4‐Ka‐22 in these mediums and PGI values against *F. solani* were determined as responses. The experimental design with 17 experiments, estimated and experimental responses obtained within the scope of the experiments are given in Table 3.

**Table 3:** RSM prepared for the optimization of the amounts of components in the production medium, experimental and estimated responses.

No.	Molasses (%) (A)	Soybean meal (%) (B)	CaCl_2_ (%) (C)	Cell growth (log (cfu/mL))		PGI (%)	
				Experimental	Estimated	Experimental	Estimated
1	0.1	1	0.75	9.4	9.73	4.49	3.23
2	1	1	0.75	9.26	8.89	7.87	7.72
3	0.1	10	0.75	8.76	8.79	8.99	9.13
4	1	10	0.75	8.08	7.96	7.87	9.13
5	0.1	5.5	0.5	9.51	9.3	8.99	9.97
6	1	5.5	0.5	8.27	8.46	10.11	9.97
7	0.1	5.5	1	9.38	9.23	2.25	2.39
8	1	5.5	1	8.12	8.39	7.87	6.88
9	0.55	1	0.5	9.24	9.355	7.87	8.15
10	0.55	10	0.5	8.16	8.41	19.1	17.98
11	0.55	1	1	9.13	9.28	7.87	8.99
12	0.55	10	1	8.25	8.34	6.74	6.46
13	0.55	5.5	0.75	9.06	8.84	5.62	4.22
14	0.55	5.5	0.75	9.15	8.84	4.49	4.22
15	0.55	5.5	0.75	8.88	8.84	3.25	4.22
16	0.55	5.5	0.75	8.94	8.84	4.49	4.22
17	0.55	5.5	0.75	8.73	8.84	3.25	4.22

Analysis of variance (ANOVA) was used to analyze the suitability of the model obtained for the number of live cells used as a response in the experimental design and the model was found to be significant. According to the results, molasses (A) and soybean meal (B) components were found to be significant to increase the number of live cells. As a result of the experiments, *R* ^2^ value was found to be 0.8182 and it was determined that the model was 81.82% suitable for increasing the cell growth. As a result of the regression analysis, Equation (2) was found, which was determined in terms of molasses amount (*A*), soybean meal amount (*B*), and CaCl_2_ amount (*C*), which are independent variables that increase the cell growth (*Y*).

(2) CellGrowh(Y)=8.84−0.424×A−0.47×B−0.036×C.

Three‐dimensional response surface graphs and perturbation graphs were examined and optimum value ranges were used for all three independent variables. As a result of the optimization of the amounts of the components in the production medium, it was determined that the amount of molasses and soybean meal in the medium was effective on the cell growth in the production of *B. subtilis* 4‐Ka‐22 (Figure S5).

When the suitability of the model obtained for the PGI values against *F. solani*, the other component used as a response in the experimental design, was examined by ANOVA analysis, it was found that the model was significant. The *R* ^2^ value was found to be 0.9503, which means that the model was 95.03% suitable to increase PGI. The optimization of molasses amount (*A*), soybean meal amount (*B*), and CaCl_2_ amount (*C*) were found to be significant to increase the PGI value (Figure S6). As a result of the regression analysis, the independent variables that increase the PGI (*Y*) value are given in Equation (3).

(3) $$\mathrm{PGI}\;\left(Y\right)=4.22+1.12\times A+1.83\times B-2.67\times C-1.12\times A\times B+1.12\times A\times C-3.09\times B\times C-3.371E-003\times A^{2}+3.09\times B^{2}+3.09\times C^{2}.$$

Based on the response surface and perturbation plots, PGI values increased with higher concentrations of molasses and soybean meal, while CaCl_2_ had a negative effect on antifungal activity. A trade‐off was observed between molasses and soybean meal concentrations, as lower levels supported higher cell growth, whereas increased concentrations promoted greater PGI values.

Considering that viable cell density is a key parameter in biofertilizer formulations, optimization efforts prioritized maximizing biomass rather than antifungal efficacy. To develop a cost‐effective production medium for *B. subtilis* 4‐Ka‐22, the concentrations of molasses, soybean meal, and CaCl_2_ were minimized, while PGI was maintained within an acceptable range. The results of the optimization process are summarized in Table 4. The formulation with a desirability score of 1.0 was selected as optimal, consisting of 0.1% molasses, 1.0% soybean meal, and 0.5% CaCl_2_.

**Table 4:** Conditioning used in production media optimization and optimized production media.

Factor		Goal		Response		Objective
Molasses (%)		Minimize		Cell growth (log)		Maximize
Soybean meal (%)		Minimize		Percent growth inhibition (PGI) (%)		In range
CaCl_2_ (%)		Minimize				

	Molasses	Soybean meal	CaCl_2_	Cell growth	PGI	Desirability
Estimated	0.10	1.00	0.50	9.76633	7.01686	1.000

Following the development of *B. subtilis* 4‐Ka‐22 in the production medium indicated in Table 4, the cell growth was 1.62 × 10^9^ cfu/mL (log 9.20) and the PGI value *against F. solani* was 16.45%. The RSM optimization of medium components revealed that the PGI value against *F. solani* was strongly influenced by the interaction between soybean meal and CaCl_2_ concentrations. Molasses exhibited a nonlinear effect on PGI; very low or high levels supported higher production, whereas intermediate concentrations reduced yield. Overall, the combination of high soybean meal and CaCl_2_ levels with an appropriate molasses concentration highlights the critical role of both nutrient availability and ion‐mediated regulation in enhancing LP production.

The addition of glycerol to the production medium is known to increase the biomass rate and support the production of surfactin on *B. subtilis* sp. (Janek et al. 2021). In order to increase the biomass rate, it was decided to add glycerol to the production medium, 1%, 2%, 5%, and 10% glycerol was added to the medium and incubation was carried out at 30°C. When the live cell counts and PGI values determined from the production media were analyzed, it was decided that the best result was obtained with 1% glycerol addition. Addition of 1% glycerol to the optimized production medium resulted in a 1.9‐fold increase in cell growth and a 2.2‐fold increase in PGI.

Mechanistically, molasses can serve as a readily available carbon source that accelerates primary metabolism and supplies precursors for LP biosynthesis; it may also provide trace elements that function as enzymatic cofactors (Zhu et al. 2012). Similarly, although not directly tested within this optimization set, glycerol can act both as a carbon source and as an osmoregulator, thereby improving cell integrity under fermentation stress and enhancing LP production (Janek et al. 2021). In addition, glycerol is an economical medium component that enables bacterial cells to better withstand abiotic stress conditions, further supporting consistent and efficient LP synthesis (Lobo et al. 2019).

In this study, aimed at achieving cost‐effective LP production, agro‐industrial wastes were employed as fermentation substrates to reduce production medium costs. However, the use of such wastes can pose challenges for ensuring consistent yields, as their natural constituents may vary in composition (Eliodório et al. 2023). Numerous studies in the literature have demonstrated successful low‐cost LP production using components such as vegetable juices and alternative carbon sources (Zhu et al. 2012; Umar et al. 2021; Gugel et al. 2024). When utilizing agro‐industrial wastes, it is essential to perform a comprehensive analysis of their basic components and to refine these compositions through statistical optimization methods. With appropriate bioprocess design and rigorous statistical control, reliable and consistent LP production can be achieved across batches.

### 3.7 Optimization of Production Parameters for *B. subtilis* 4‐Ka‐22

The media formulation was finalized as 0.1% molasses, 1% soybean meal, 0.50% CaCl_2_, and 1% glycerol after determining the amount of glycerol to be added to the production medium. After the determination of the content, the optimization of the media parameters was started and temperature optimization experiments were carried out first. In this context, temperatures of 30°C, 33°C, 36°C, and 40°C were tested. Cell growth and PGI data for the temperature experiments are given in Table 5. When cell growth and PGI values are examined, the highest was observed at 30°C in the optimized production medium containing glycerol, while the highest PGI value was observed at 33°C. Since there was no statistically significant difference between the PGI values according to ANOVA analysis, the optimum production temperature was selected as 30°C considering cell growth and low energy costs in the production medium with glycerol.

**Table 5:** Effect of different temperature values on live cell count and PGI.

Temperature	Cell Growth ± SD^a^ (×10^9^ cfu/mL)	PGI ± SD (%)
30°C	2.89 ± 0.23^a^	8.37 ± 1.13^a^
33°C	2.58 ± 0.43^a^	10.9 ± 1.72^a^
36°C	1.49 ± 0.06^b^	7.74 ± 1.21^a^
40°C	1.07 ± 0.35^b^	7.03 ± 1.21^a^

*Note:* Means within the columns with the same letter are not significantly different according to Tukey's test (*p* < 0.05).

^a^ SD, standard deviation.

Optimization of the temperature parameter was followed by RSM analysis and Box–Behnken experimental design to optimize the shaker stirrer speed, pH, and inoculation rate. The parameters of cell growth (cfu/mL) and antifungal effect (PGI) were used as response. The data obtained as a result of the development of the *B. subtilis* 4‐Ka‐22 in the optimized production medium using the specified parameters in the 17‐experiment design generated by the program are given in Table 6. In order to obtain high biomass, the suitability of the model was examined using ANOVA, and the data predicted by the program and obtained as a result of the experiments are given in Table 6.

**Table 6:** RSM prepared for optimization of production parameters, experimental and estimated responses.

No.	Shaker speed (rpm) (A)	pH (B)	Inoculation (%) (C)	Cell growth (log (cfu/mL))		PGI (%)	
				Experimental	Estimated	Experimental	Estimated
1	150	5	3	9.04	9.06	5.56	6.39
2	210	5	3	9.16	9.13	12.22	11.11
3	150	9	3	9.07	9.1	3.33	4.44
4	210	9	3	8.91	8.89	3.33	2.5
5	150	7	1	8.85	8.83	11.11	9.58
6	210	7	1	8.77	8.71	7.78	8.19
7	150	7	5	9.11	9.18	3.33	2.92
8	210	7	5	8.94	9.06	5.56	7.08
9	180	5	1	8.81	8.91	11.11	11.81
10	180	9	1	8.65	8.75	5.56	5.97
11	180	7	5	9.33	9.24	7.78	7.36
12	180	7	5	9.31	9.21	3.33	2.64
13	180	7	3	9.27	9.22	7.78	10.22
14	180	7	3	9.17	9.22	12.22	10.22
15	180	7	3	9.28	9.22	12.22	10.22
16	180	7	3	9.11	9.22	8.89	10.22
17	180	7	3	9.26	9.22	10	10.22

ANOVA analysis of the experimental design revealed that the model developed to evaluate the effect of selected parameters on cell growth was statistically significant. Among the variables, increasing the inoculation rate (*C*, *C* ^2^) and shaker speed (*A* ^2^) had a significant positive effect on viable cell counts. The coefficient of determination (*R* ^2^) was calculated as 0.8591, indicating that the model could explain 85.91% of the variation in cell growth. This suggests that the selected model is capable of adequately describing the second‐order effects of the tested variables on biomass increase. Based on the regression analysis, Equation (4) was derived to represent the relationship between viable cell count (*Y*) and the independent variables: shaker speed (*A*), pH (*B*), and inoculation rate (*C*).

(4) $$\mathrm{Cell}\;\mathrm{Growth}\;\left(Y\right)=10.22+0.69\times A-2.64\times B-1.94\times C-1.67\times A\times B+1.39\times A\times C+0.28\times B\times C-2.06\times A^{2}-2.06\times B^{2}-1.22\times C^{2}.$$

The three‐dimensional response surface and perturbation plots indicate that PGI decreases at both lower and higher shaker speeds, while it increases with decreasing pH and inoculation rate (Figures S7 and S8). The influence of pH and inoculation rate on PGI is notably greater than that of shaker speed. All three variables were optimized within defined ranges, and the results clearly demonstrate that pH and inoculation rate are the most influential factors affecting PGI during production with *B. subtilis* 4‐Ka‐22. After the validation studies, the production parameters were 195 rpm for shaker speed, 5.0 for pH value, and 4.33% for inoculation rate. As a result of the experiments, the cell growth and PGI values of *B. subtilis* 4‐Ka‐22 against *F. solani* in different media and production mediums are given in Table 7.

**Table 7:** Cell growth and PGI obtained in the optimization stages.

	Cell growth ± SD^a^ (×10^8^ cfu/mL)	PGI ± SD (%)
LB broth medium (control)	2.44 ± 0.6	13.04 ± 2.2
Optimized production medium	16.2 ± 3.8	16.45 ± 3.0
Optimized production medium + glycerol	28.9 ± 2.3	8.37 ± 1.1
Production parameters after optimization	32.7 ± 3.7	8.04 ± 1.7

^a^ SD, standard deviation.

## 4 Conclusion

Production medium and parameter optimization was carried out for the selected *B. subtilis* 4‐Ka‐22 strain after the initial screening of *Bacillus* isolates. The optimized medium resulted in a viable cell count of 3.27 × 10^9^ CFU/mL, representing a 13.4‐fold increase compared to the count obtained in standard LB medium (2.44 × 10^8^ CFU/mL). The use of agro‐industrial wastes in the formulation significantly reduced production costs, making the process economically favorable. When compared to LB broth, the optimized medium achieved a cost reduction of more than 99% on a per‐liter basis, highlighting its potential for large‐scale, low‐cost production. Overall, this study provides a valuable example of cost‐effective medium optimization for the production of LPs with potential applications in various industrial and biotechnological fields. These findings underscore the feasibility of integrating statistical optimization with low‐cost substrates to enhance both yield and economic viability.

## Author Contributions

**Derya Maral‐Gül:** investigation, methodology, data curation, formal analysis, visualization. **Zülal Günay:** investigation, visualization, writing – original draft, review, and editing. **Baran Mis:** writing – review, and editing. **Rengin Eltem:** resources, supervision, validation, conceptualization, project administration, writing – review and editing.

## Ethics Statement

The authors have nothing to report.

## Conflicts of Interest

The authors declare no conflicts of interest.

## Supporting information


**Figure 1:** Classification of hemolytic activity of *Bacillus* isolates (9–13 mm low hemolytic activity, 13–17 mm medium hemolytic activity, 17–21 mm high hemolytic activity) and hemolysis zones formed on blood agar medium A) *B. subtilis* 4‐Ka‐22, B) *S. aureus* BAA‐40. **Figure 2:** Effect of different carbon sources on cell growth (cfu/ml) and PIG value as a result of LP production in *B. subtilis* 4‐Ka‐22. **Figure 3:** Effect of different nitrogen sources on cell growth (cfu/ml) and PIG value as a result of LP production in *B. subtilis* 4‐Ka‐22. **Figure 4:** Effect of different production medium components on cell growth (cfu/ml) and PIG value as a result of LP production in *B. subtilis* 4‐Ka‐22. **Figure 5:** 3D response surface plots obtained as a result of RSM optimization of production medium content A. Effect of changing the amount of molasses and soybean meal on the cell growth B. Effect of changing the amount of CaCl₂ and soybean meal on the cell growth C. Effect of changing the amount of CaCl₂ and molasses on the cell growth. **Figure 6:** 3D response surface plots obtained as a result of RSM optimization of production medium content A. effect of varying the amount of molasses and soy flour on PGI B. effect of varying the amount of molasses and CaCl₂ on GCI C. effect of varying the amount of soy flour and CaCl₂ on PGI. **Figure 7:** 3D surface response graphs obtained as a result of RSM optimization of production medium parameters A. Effect of rpm and pH changes on viable cell number B. Effect of rpm and inoculation rate on viable cell number C. Effect of pH and inoculation rate on viable cell number. **Figure 8:** 3D surface response graphs obtained as a result of RSM optimization of production parameters A. Effect of rpm and pH changes on PGI value B. Effect of rpm and inoculation rate on GCI value C. Effect of pH and inoculation rate on PGI value. **Table 1:** Determination of surfactin and iturin‐A amounts of *Bacillus* sp. screened in the study by Q‐TOF.

## Data Availability

The data that support the findings of this study are available in the Supporting Material of this article.
