# Supplementary material for: An Economical Bioprocess for Lipopeptide Production From Bacillus subtilis
Source: Microbiologyopen. 2026 May 8;15(3):e70130. doi: 10.1002/mbo3.70130 (PMC13154768; doi:10.1002/mbo3.70130)
Supplement: Supplementary file 1 — Figure 1: Classification of hemolytic activity of Bacillus isolates (9–13 mm low hemolytic activity, 13–17 mm medium hemolytic activity, 17–21 mm high hemolytic activity) and hemolysis zones formed on blood agar medium A) B. subtilis 4‐Ka‐22, B) S. aureus BAA‐40. Figure 2: Effect of different carbon sources on cell growth (cfu/ml) and PIG value as a result of LP production in B. subtilis 4‐Ka‐22. Figure 3: Effect of different nitrogen sources on cell growth (cfu/ml) and PIG value as a result of LP production in B. subtilis 4‐Ka‐22. Figure 4: Effect of different production medium components on cell growth (cfu/ml) and PIG value as a result of LP production in B. subtilis 4‐Ka‐22. Figure 5: 3D response surface plots obtained as a result of RSM optimization of production medium content A. Effect of changing the amount of molasses and soybean meal on the cell growth B. Effect of changing the amount of CaCl₂ and soybean meal on the cell growth C. Effect of changing the amount of CaCl₂ and molasses on the cell growth. Figure 6: 3D response surface plots obtained as a result of RSM optimization of production medium content A. effect of varying the amount of molasses and soy flour on PGI B. effect of varying the amount of molasses and CaCl₂ on GCI C. effect of varying the amount of soy flour and CaCl₂ on PGI. Figure 7: 3D surface response graphs obtained as a result of RSM optimization of production medium parameters A. Effect of rpm and pH changes on viable cell number B. Effect of rpm and inoculation rate on viable cell number C. Effect of pH and inoculation rate on viable cell number. Figure 8: 3D surface response graphs obtained as a result of RSM optimization of production parameters A. Effect of rpm and pH changes on PGI value B. Effect of rpm and inoculation rate on GCI value C. Effect of pH and inoculation rate on PGI value. Table 1: Determination of surfactin and iturin‐A amounts of Bacillus sp. screened in the study by Q‐TOF. [file MBO3-15-e70130-s001.docx]

**SUPPLEMENTARY MATERIALS**

**Table 1.** Determination of surfactin and iturin-A amounts of *Bacillus* sp. screened in the study by Q-TOF

| *Bacillus* isolate | Iturin A ±SD*  (μg/ml) | Surfactin ±SD (μg/ml) | *Bacillus* isolate | Iturin A ±SD (μg/ml) | Surfactin ±SD (μg/ml) |
| --- | --- | --- | --- | --- | --- |
| *T-1-18* | 16,94±0,4 | 7,89±1,7 | ***Ö-5-1*** | 20,5±2,8 | 33±12,7 |
| *T-18-a* | 17,28±0,8 | 6,64±0,1 | ***K-6-22*** | 13,4±5,4 | 28,3±9,2 |
| *T-2-2* | 31,09±1,8 | 12,77±1,1 | ***K-9-37-a*** | 32,7±0,7 | 40,1±2,6 |
| *T-2-16* | 31,05±2,3 | 12,55±1,4 | ***4-Ka-22*** | 31,9±0,4 | 41,9±3,5 |
| *T-3-10* | ND | 13,91±0,2 | ***A-1*** | 32,5±0 | 35,6±8,4 |
| *T-4-8* | 32,27±2,9 | 12,28±0,5 | ***Ö-2-55-a*** | 16,01±2,7 | 6,97±0,04 |
| *T-4-9* | 28,82±0,6 | 12,41±1,7 | ***Ö-4-57-b*** | 11,51±0,3 | 10,84±1,7 |
| *T-4-13-a* | 25,47±1,9 | 14,05±1,5 | ***T-3-12-a*** | 11,34±1,7 | 6,62±0,1 |
| *T-4-14-a* | 45,39±13 | 12,94±1,4 | ***T-3-12-b*** | 19,57±1,3 | 10,42±0,2 |
| *T-4-14-b* | 30,73±2,5 | 10,93±6,9 | ***T-4-20-e-a*** | 13,13±1,9 | ND |
| *T-4-15* | 27,69±1,9 | 14,29±2,3 | ***Ç-3-20-a*** | 15,57±0,6 | 10,97±0,3 |
| *T-4-17* | ND | 10,16±2 | ***K-7-14-1*** | 13,66±0,9 | 6,56±0,02 |
| *T-4-19* | ND | 3,06±3,9 | ***1-K-49-a*** | 13,9±0,9 | 10,6±0,5 |
| *T-11-8* | 23,55±0,4 | 11,84±6,1 | ***2-K-5*** | 10,07±1 | 7,36±0,06 |
| *Ç-2-30-a* | 18,37±0,9 | 7,12±0,3 | ***2-K-13-a*** | 14,08±0,4 | 7,13±0,7 |
| *Ç-2-30-b* | 28,99±0,2 | 10,93±2,9 | ***2-K-29*** | 36,23±2,6 | 9,47±1,4 |
| *Ç-2-36* | ND | 12,53±1,4 | ***2-K-35-a*** | 27,25±7,3 | 8,79±1,3 |
| *Ç-3-10* | 33,46±0,8 | 12,77±1,4 | ***2-K-37*** | 9,82±0,6 | 10,17±1 |
| *Ç-3-15-a* | 38,18±2,8 | 10,23±0,2 | ***3-K-10*** | 20,38±6,2 | ND |
| *Ç-3-19* | ND | 13,05±0,7 | ***3-K-25*** | 10,08±0,6 | 7,5±0,04 |
| *Ç-3-22-a* | 29,4±4,7 | 12,8±8,5 | ***4-Ka-59-a-a*** | 38,15±6,5 | 8,6±0,4 |
| *Ç-3-23* | 22,1±0 | 23±13,5 | ***3-K-S-1-a*** | 11,7±2,9 | 8,99±1,2 |
| *Ç-3-31* | 5,9±0 | 35,4±13,1 | ***3-K-S-14*** | ND | ND |
| *Ö-1-31-b* | 27,4±3,9 | 26,5±16,1 | ***3-K-S-17-a*** | 16,01±0,04 | ND |
| *Ö-1-57-b* | 1,0±0 | 34±4,3 | ***3-K-S-37*** | 12,99±3,4 | 8,91±0,8 |
| *Ö-1-59-b* | 32,7±0,9 | 29±13,2 | ***3-K-S-39-a*** | 10,38±0,2 | 6,88±0,2 |
| *Ö-1-85* | 32±0,1 | 38,2±4 | ***3-K-S-47-a*** | 11,51±0,1 | 7,09±0,8 |
| *Ö-2-44-b* | 15,5±0 | 29,4±5,7 | ***3-K-S-49*** | 9,1±0,2 | 10,64±0,2 |
| *Ö-2-61-a* | 29,6±0 | 25,4±2,6 | ***3-K-S-60-a*** | 15,99±5,7 | 8,34±1,1 |
| *Ö-3-5-b* | 0± | 28,3±2,9 | ***3-K-S-61*** | 12,16±0,6 | 7,31±0,4 |
| *Ö-3-21* | 33,3±0 | 35±13,7 | ***3-K-S-64*** | 15,82±0,5 | 9,43±2,2 |
| *Ö-4-11-a* | 0± | 41±3,1 | ***K-6-13-b-a*** | 13,81±0,9 | 7,95±0,1 |
| *Ö-4-13-a* | 0± | 39,8±3,4 | ***Ö-6-50-a*** | 14,77±2,1 | 11,16±1,4 |
| *Ö-4-68* | 28±10,4 | 31,2±10,7 | ***Ç-1-16-a*** | 14,76±0,5 | 9,61±1,6 |
| *Ö-4-82* | 0± | 25,5±10 |  |  |  |

*SD: Standard Deviation ND: Not detected


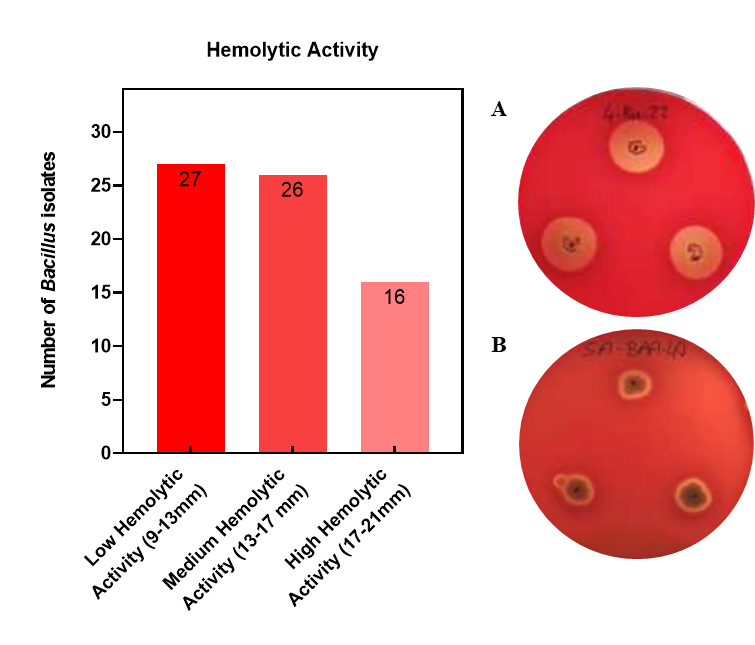


**Figure 1.** Classification of hemolytic activity of *Bacillus* isolates (9-13mm low hemolytic activity, 13-17mm medium hemolytic activity, 17-21mm high hemolytic activity) and hemolysis zones formed on blood agar medium **A)** *B. subtilis* 4-Ka-22, **B)** *S. aureus* BAA-40

In order to examine the effect of the components to be used in the optimization of the production medium, the effect of the addition of LB (Bysogeny Broth) medium on the number of viable cells and PGI (Figures 2, 3 and 4).

**Figure 2.** Effect of different carbon sources on cell growth (cfu/ml) and PIG value as a result of LP production in *B. subtilis* 4-Ka-22

**Figure 3.** Effect of different nitrogen sources on cell growth (cfu/ml) and PIG value as a result of LP production in *B. subtilis* 4-Ka-22

**Figure 4.** Effect of different production medium components on cell growth (cfu/ml) and PIG value as a result of LP production in *B. subtilis* 4-Ka-22


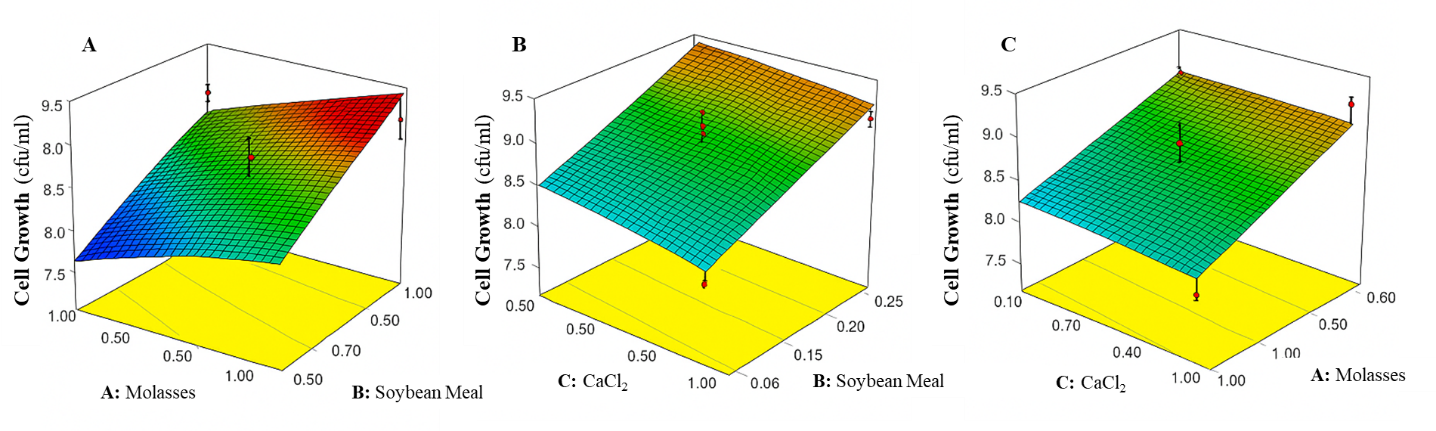


**Figure 5.** 3D response surface plots obtained as a result of RSM optimization of production medium content **A**. Effect of changing the amount of molasses and soybean meal on the cell growth **B.** Effect of changing the amount of CaCl_2_ and soybean meal on the cell growth **C.** Effect of changing the amount of CaCl_2_ and molasses on the cell growth


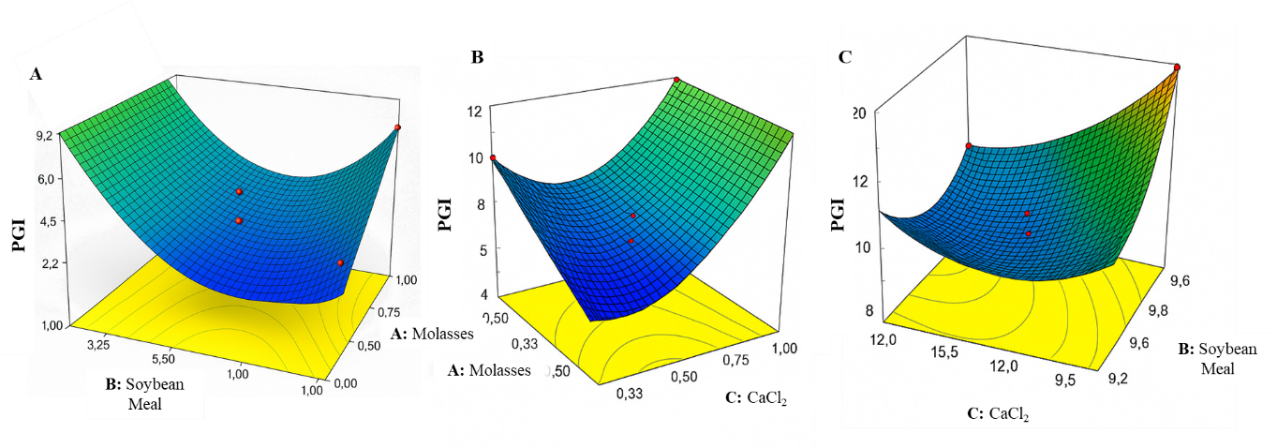


**Figure 6.** 3D response surface plots obtained as a result of RSM optimization of production medium content A. effect of varying the amount of molasses and soy flour on PGI B. effect of varying the amount of molasses and CaCl_2_ on GCI C. effect of varying the amount of soy flour and CaCl_2_ on PGI


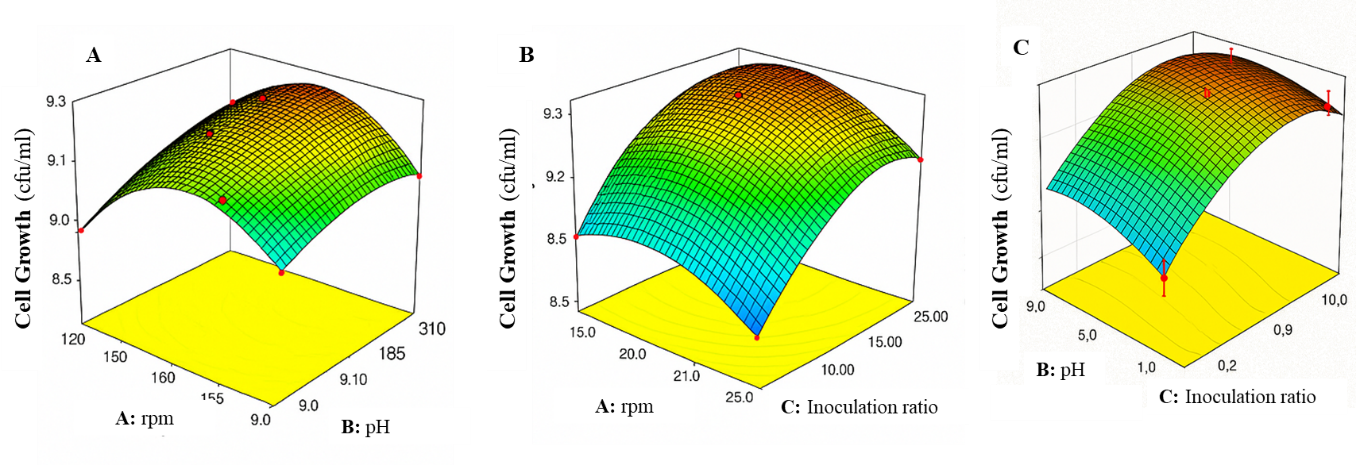


**Figure 7.** 3D surface response graphs obtained as a result of RSM optimization of production medium parameters A. Effect of rpm and pH changes on viable cell number B. Effect of rpm and inoculation rate on viable cell number C. Effect of pH and inoculation rate on viable cell number


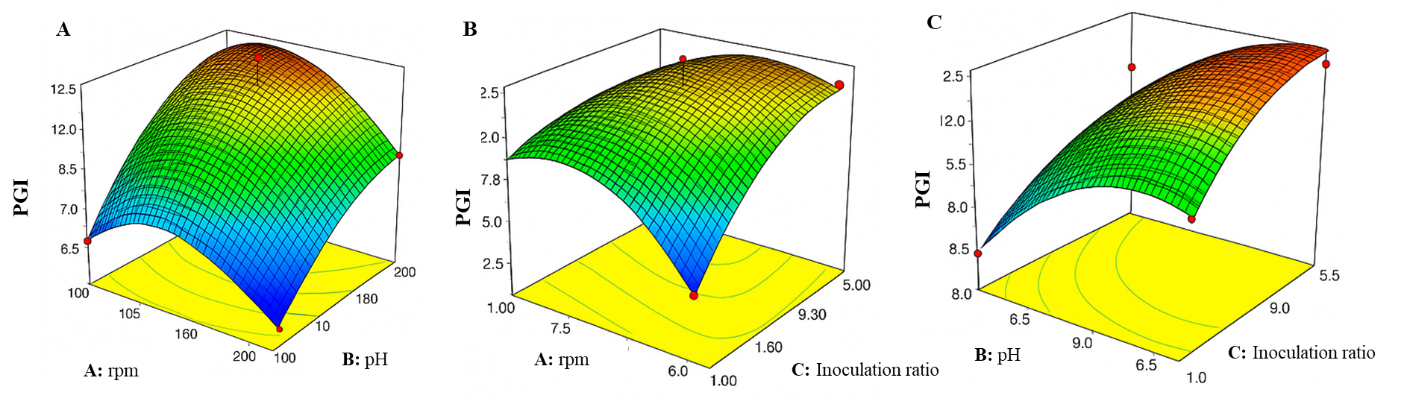


**Figure 8.** 3D surface response graphs obtained as a result of RSM optimization of production parameters A. Effect of rpm and pH changes on PGI value, B. Effect of rpm and inoculation rate on GCI value C. Effect of pH and inoculation rate on PGI value
